# Cutting-Edge Technology Without Cutting: Treating Skin Cancer in This Era—A Case Series

**DOI:** 10.3390/cancers16213557

**Published:** 2024-10-22

**Authors:** Babar Rao, Alexis Moreno, Muhammad Shahmir Abbasi, Noah Musolff, Bianca Sanabria, Vlad Voiculescu

**Affiliations:** 1 Rao Dermatology, New York, NY 10003, USA; 2 Rutgers Center for Dermatology, Somerset, NJ 08873, USA; 3 Department of Dermatology, Weill Cornell Medicine, New York, NY 10021, USA; 4 Rao Dermatology, Fresno, CA 93720, USA; 5 Department of Human Nutritional Sciences, California State University, Fresno, CA 93740, USA; 6 Department of Internal Medicine, Dartmouth Hitchcock Medical Center, Lebanon, NH 03766, USA; 7 Rutgers Robert Wood Johnson Medical School, Rutgers University, New Brunswick, NJ 08901, USA; 8 Department of Oncological Dermatology, Carol Davila University of Medicine and Pharmacy, 050474 Bucharest, Romania; 9 Elias Emergency University Hospital, 011461 Bucharest, Romania

**Keywords:** non-melanoma skin cancer, reflectance confocal microscopy, superficial radiation therapy, non-invasive management

## Abstract

**Simple Summary:**

This case series study evaluates the use of reflectance confocal microscopy (RCM) for assessing treatment outcomes after superficial radiation therapy (SRT) in managing localized non-melanoma skin cancer (NMSC). Conducted between March 2020 and December 2023, this study included 29 patients with 38 lesions. Following RCM diagnosis, the patients underwent SRT, and outcomes were assessed at six months using RCM and clinical evaluation. The results showed 100% tumor clearance with no residual activity observed upon conducting follow-up RCM. Clinically, scarring and mild erythema were observed, with moderate to severe inflammation in six lesions. While the generalizability of the results of our study is limited by the size of the study population, our findings suggest that RCM and SRT may be effective in managing localized NMSC non-invasively, potentially offering an alternative to traditional surgical methods, particularly for elderly or surgically unsuitable patients.

**Abstract:**

Background: Traditional treatment methods for non-melanoma skin cancer (NMSC) include surgical excision with histological evaluation, yet advancements such as reflectance confocal microscopy (RCM) and superficial radiation therapy (SRT) offer non-invasive management alternatives. This study aims to evaluate the use of RCM for the evaluation of treatment outcomes after SRT in managing localized NMSC. Methods: A prospective interventional case series study was conducted on patients treated for NMSC with SRT between March 2020 and December 2023. Suspected NMSC lesions were initially evaluated with a handheld dermoscope and then imaged at multiple depths using a VivaScope 1500 RCM. Two dermatologists trained in RCM reviewed the images. Confirmed NMSC lesions were biopsied and treated with SRT, followed by RCM imaging at six months post-treatment to assess cancer clearance, scarring, and inflammation. Results: Of the 38 lesions (composed of SCC (24) and BCC (14)) treated affecting the 29 patients, all lesions showed no residual tumor activity upon conducting follow-up RCM (100% clearance). Scarring and mild erythema were noted clinically. Six lesions demonstrated moderate to severe inflammation at a 6-month follow-up. Conclusions: This study demonstrates successful non-invasive management of localized NMSC using RCM and SRT. RCM was able to non-invasively demonstrate complete tumor clearance achieved by SRT with minimal adverse effects. These findings support considering the use of RCM and SRT as primary diagnostic, monitoring, and treatment options for NMSC without the need for biopsies, especially for elderly patients or those unsuitable for surgery due to medical conditions.

## 1. Introduction

Non-melanoma skin cancers (NMSCs) are the most common type of malignancy worldwide, with a 4:1 split between basal cell (BCC) versus cutaneous squamous cell carcinoma (SCC) [1]. In the United States alone, 5.4 million cases of NMSC are diagnosed each year, a number which is suspected to be an underrepresentation, given how frequently they can remain undiagnosed before disease progression [1,2]. Although mortality rates for BCC and SCC are low, these cancers can cause significant morbidity due to disfigurement, as the lesions commonly appear on the skin of the head and neck. There are several critical risk factors associated with the development of NMSC, and the most important ones include old age; chronic ultraviolet (UV) light exposure, predominantly from the sun; and lighter skin tones [1,2].

Many advancements have been made toward the diagnosis, management, and monitoring of NMSC. In vivo reflectance confocal microscopy (RCM) is an established method that allows for the non-invasive diagnosis of cutaneous lesions, including BCC, with a sensitivity of up to 97% and a specificity of 93%, when used in conjunction with dermoscopy [3,4]. The major diagnostic criteria for BCC in regard to RCM include polarized elongated features, basaloid nodules, epidermal shadowing/dark clefts, and telangiectasias [5,6]. In an RCM analysis, invasive SCC displays an irregular honeycomb architecture and speckled nucleated atypical cells in the dermis [7]. RCM also allows for the distinction between actinic keratosis and invasive SCC based on the presence of architectural disarray in the stratum spinosum and granulosum, with a positive predictive value of 88.5% [8].

The National Comprehensive Cancer Network (NCCN) recognizes several invasive treatment methods for BCC, including curettage, electrodessication, traditional surgical excision, and Mohs micrographic surgery, which is considered the gold standard [9]. The non-invasive localized therapies comprise topical imiquimod, cryotherapy, and photodynamic therapy, which are usually used to treat superficial lesions. Systemic therapies with Hedgehog pathway inhibitors and PD-1 inhibitors show promise for locally advanced and metastatic lesions [10,11,12]. Radiation therapy (RT) is recommended as an alternative therapy for advanced BCC, being a primary choice for non-surgical candidates. It significantly benefits the geriatric patient population, who often have medical comorbidities that make surgery difficult or simply wish to avoid an invasive procedure [13]. The five-year rate of recurrence after RT varies from 4% to 16%, likely due to its traditional use as a first-line treatment for advanced, especially metastatic, disease [9].

SCC has localized treatment options similar to those for BCC, with Mohs surgery being the gold standard and the inclusion of topical 5-fluorouracil serving as a noninvasive therapy alternative [14]. Due to the higher risk of invasion and metastasis, RT has been established as a treatment option for higher-staged disease. The NCCN 2021 recommendations for cutaneous SCC also suggest that patients below the age of 60 should be allowed to receive superficial RT for limited-stage disease, which was previously suggested to be used primarily for older patients for multiple reasons, including personal preference [15].

While histological evaluation and surgical excision have been established as the gold standard for the diagnosis and treatment of NMSC, non-invasive methods have also been validated for these roles. Therefore, in our study, we assessed the effectiveness of combining in vivo RCM and superficial radiation therapy (SRT) for the complete non-invasive management of SCC in situ, invasive SCC, and superficial and nodular BCC lesions.

## 2. Materials and Methods

We conducted a prospective interventional case series study of patients from a private dermatology practice in Fresno, California, who received superficial radiation therapy (SRT) between March 2020 and December 2023. Our inclusion criteria specified the admission of patients aged 18 and above who could provide written informed consent and wished to undergo superficial radiation therapy for the treatment of NMSC, irrespective of cancer type. Patients with lesions on mucosal surfaces, palmoplantar areas of the body, or areas difficult to image with RCM; patients that were undergoing pharmacological treatment for NMSC at the time of the study; and patients who did not complete their SRT sessions were excluded from this study. Each lesion on a patient was treated as an individual NMSC. All subjects gave their written informed consent for inclusion before they participated in this study. This study was conducted in accordance with the Declaration of Helsinki, and the protocol was approved by Advara (Pro00035376).

### 2.1. Superficial Radiation Therapy

SRT was administered using the FDA-approved Sensus SRT-100 Vision™ (SkinCure Oncology, Burr Ridge, IL, USA). This device uses a built-in high-frequency ultrasound system to evaluate tumor depth and extent, which enables calculation of the necessary total dose and non-invasive monitoring of treatment progression. To limit the radiation dose in each session, a daily dose of 265–278 cGy, with a typical number of 20 treatment sessions, was applied. In two cases, 22 sessions were needed due to remaining tumor activity observed via ultrasound imaging. The sessions were split into 3 fractions per week, with an average treatment duration of seven weeks. The mean total radiation dose was 5443.8 to 5640.8 cGy (mean: 5522.2 cGy; median: 5510.4 cGy; SD: 42.37 cGy).

### 2.2. Diagnosis Using RCM

Each lesion was evaluated with a handheld dermoscope using established diagnostic criteria, including structureless areas (SCC), looped and/or arborizing vessels (SCC/BCC), and leaf-like areas (BCC). Lesions suspicious for NMSC diagnosis were then imaged using a fourth-generation VivaScope 1500 reflectance confocal microscope (Caliber I.D, Rochester, NY, USA). Prior to imaging, the area of interest was cleaned and/or shaved as needed. Images were captured as VivaCubes at four different depths: sub-stratum corneum, stratum spinosum, dermoepidermal junction, and superficial dermis. A Vivastack of each target lesion was taken as well. All images were then assessed by two dermatologists trained in RCM imaging, who made the final diagnosis based on BCC and SCC criteria in the literature [16,17]. Benign or ambiguous lesions were excluded. After RCM imaging was conducted, the lesions underwent biopsies for histological confirmation of NMSC presence and type. The patients then underwent their SRT treatment sessions. Six months post-treatment, a follow-up RCM was performed to confirm skin cancer clearance and evaluate the extent of scarring and remaining inflammation. Inflammation was graded based on the presence of inflammatory cells: none (<5 cells/image), mild (5–10 cells/image), moderate (11–15 cells/image), or severe (>20 cells/image).

### 2.3. Data Analysis

Patient demographic information, including age, gender, ethnicity, and history of skin cancer, was collected. Tumor diagnosis, location, size, SRT dose, and degree of inflammation post-treatment were also included as part of the data analysis. Simple descriptive statistics obtained using Microsoft Excel were used to assess overall trends.

## 3. Results

**Figure 1:**
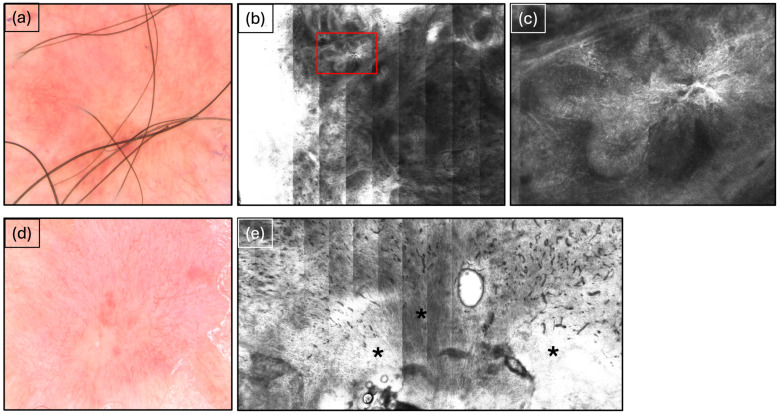
Basal cell carcinoma before and after SRT. (**a**) Basal cell carcinoma imaged via dermoscopy before SRT showing characteristic arborizing vessels. (**b**) Basal cell carcinoma observed via reflectance confocal microscopy (RCM) before superficial radiation therapy (SRT). (**c**) Red cutout from (**b**) showing characteristic tumor islands. (**d**) Appearance in dermoscopy after SRT at the 6-month follow-up showing erythema and scarring evident in the treatment area. (**e**) Appearance in RCM after SRT at 6-month follow-up shows extensive scarring (black asterisks).

**Figure 2:**
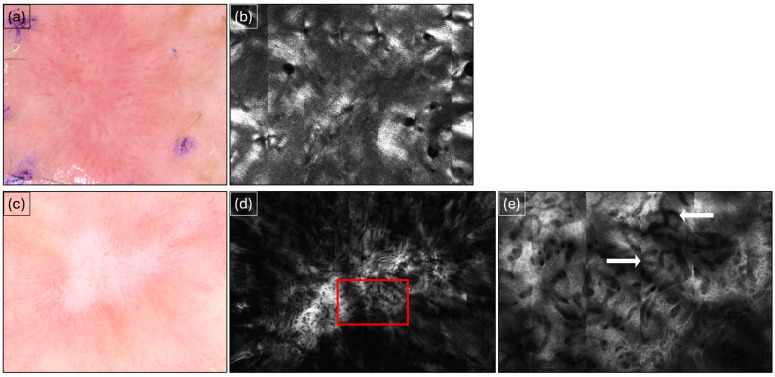
Squamous cell carcinoma before and after SRT. (**a**) Squamous cell carcinoma observed via dermoscopy before superficial radiation therapy (SRT) showing characteristic dotted vessels and white structureless areas. (**b**) Squamous cell carcinoma imaged via reflectance confocal microscopy (RCM) before SRT showing irregular honeycombing. (**c**) Appearance in dermoscopy after SRT at the 6-month follow-up showing erythema and scarring evident in the treatment area. (**d**) Appearance in RCM after SRT at 6-month follow-up. (**e**) Red cutout from (**d**) showing extensive scarring and numerous blood vessels (white arrows).

A total of 29 patients were recruited for this study, comprising 20 males (68.97%) and 9 females (31.03%), with a combined total of 38 lesions. A maximum of three lesions in the same patient were treated. The mean age was 70.5 ± 12.4 years, and 97% of the patients were Caucasian. Table 1 summarizes the basic patient characteristics and demographics. The most common lesions were SCC, with 24 cases (63%), of which 6 corresponded to SCC in situ (16%), followed by 14 corresponding to BCC (37%), of which 10 were nodular (26%) and 4 (11%) were superficial subtypes. Six patients (and six lesions) were lost to follow-up. Of the 32 lesions fully treated, no lesions showed residual tumor activity in follow-up RCM imaging (100% clearance, Table 2, Figure 1 and Figure 2). The patients received a mean radiation dose of 5522.2 ± 42.37 cGy (with a range from 5443.8 to 5640.8 cGy) over the course of 7 weeks, with an average of three treatment sessions per week. Clinically, after 6 months, all lesions showed scarring limited to the treatment area with surrounding mild erythema. No adverse effects were observed. Upon conducting RCM imaging, six of the lesions (16%) showed moderate to severe inflammation six months after the patients had completed SRT.

## 4. Discussion

The results of our study are limited by its small sample size, which hinders generalizability and suggests the need for replication on a larger scale. Additionally, the comparatively short follow-up period of 6 months limits the evaluation of lesion recurrence. Studies with longer patient follow-ups are needed to assess the efficacy and duration of progression-free survival among patients with NMSC treated with SRT. However, our small study demonstrates a completely noninvasive approach to managing NMSC. At the six-month follow-up, no remaining or recurrent disease activity was detected via RCM, resulting in a 100% clearance rate. Traditionally, the treatment outcomes of surgical and non-invasive modalities for NMSC are judged based on clinical assessment, but the extent of local scarring can complicate this evaluation. The criteria for the dermoscopic diagnosis of recurrent BCC include pigmentation, superficial telangiectasias, and structureless areas, but these features can be challenging to distinguish from scar tissue, particularly in fair skin [16].

RCM has demonstrated high accuracy for the non-invasive diagnosis and monitoring of NMSC and is increasingly recognized as a potentially superior method to clinical evaluation for detecting primary NMSC. RCM non-invasively enables real-time, high-resolution imaging of the epidermis and papillary dermis at the cellular level, with magnification similar to that of ×30 histological examination. It captures optical sections that are 3 to 5 µm thick, with a lateral resolution of 0.5 to 1.0 µm, generating 500 × 500 µm^2^ images at depths of up to 200 µm [17,18,19]. These individual images can be seamlessly stitched together using specialized software to form a mosaic, expanding the view to an area as large as 8 × 8 mm^2^. Much like dermoscopy, the imaging is performed in enface planes, parallel to the skin surface. Using these capabilities, RCM allows the characterization of the healthy epidermis, dermoepithelial junction (DEJ), and upper dermis both at an architectural and cellular level. The DEJ is particularly important because many disease processes in dermatology can be identified at this level. In NMSC, the normal appearance of the skin is disturbed, and identifying the individual changes through RCM can lead us to a non-invasive diagnosis of the NMSC subtype. In BCC specifically, the main features for diagnosis consist of characteristic tumor nests (aggregations of basaloid cells) with peripheral palisading, branch-like structures, and fibrotic septa. While dermoscopy offers high diagnostic accuracy for BCC by identifying features like arborizing vessels, the utility of RCM is evident when used in clinically unequivocal cases [20,21]. A study by Ruini et al. demonstrated that RCM can successfully diagnose BCC by identifying tumor islands when evidence from dermoscopy is unclear [21]. Additionally, RCM has been shown to aid in distinguishing BCC subtypes, as tumor nests with peripheral palisading, branch-like structures, fibrotic septa, and increased vascular diameter are the main characteristic RCM features for nodular and micronodular BCC, while solar elastosis and tumor nests located just below or in connection with the basal cell layer characterize superficial BCC [22]. Furthermore, RCM has also been proven useful in demonstrating BCC recurrence in the follow up of lesions previously treated with surgical or non-surgical methods [23]. Finally, a metanalysis summarizing the results of six studies on the use of RCM in diagnosing primary BCC demonstrated high sensitivity and specificity (97% and 93%, respectively) [24]. Similarly, the criteria for the diagnosis of invasive SCC via RCM have been established and include the presence of polymorphic vessels, ulceration, architectural disarrangement, speckled nucleated cells in the dermis, irregularly dilated vessels, and the absence of hyperkeratosis [7]. In a healthy epidermis, the keratinocytes are arranged in what is called the typical honeycombing pattern, which is revealed by the enface view in RCM and consists of cells symmetrically arranged in a grid. In cutaneous SCC, among other skin lesions, this pattern is disrupted, leading to the formation of atypical honeycombing showing an irregular grid and pleomorphic cells, which is also an important clue in the diagnosis of actinic keratosis [25]. The degree of this atypia has also been used to grade its severity, a process similar to grading in histopathology [26,27]. Moreover, the next step in utilizing RCM’s ability to assess tumor features is the non-invasive monitoring of treatment success. For actinic keratosis, RCM has successfully been used during treatment with imiquimod, proving its ability to non-invasively demonstrate tumor clearance [28]. Similarly, the microscopic effects of imiquimod on BCC have been studied, where RCM assessments correlated well with tumor response to therapy [29]. However, the widespread adoption of RCM outside academic settings faces challenges due to the extensive training required, which can be difficult for new learners. Simplifying the diagnostic language and employing a more straightforward, stepwise approach have proven effective in helping novice users diagnose melanotic and NMSC with 92% sensitivity and 67% specificity based on four key diagnostic features [30]. In our study, we establish the utility of RCM in assessing ongoing or recurrent tumor activity after the treatment of NMSCs, positioning it as a viable alternative to traditional clinical follow-ups.

Exploring non-surgical treatment options for NMSCs is essential, particularly for patients who may not be suitable for surgery due to age, comorbidities, or an increased risk of poor wound healing. These less invasive alternatives offer the advantages of reduced recovery time, minimized scarring, and improved quality of life.

The findings of our study, which utilized clinical assessment and RCM to confirm tumor clearance of SCC and BCCs following SRT, align with those in the existing literature, including a study by Roth et al., which reported a 97.4% cure rate for both BCC and SCC lesions in the lower limbs that were treated with SRT in a similar patient population [31]. Further support comes from a study by Yu L et al., which demonstrated a 99.3% disease control rate through the image-guided superficial radiation therapy of 2917 limited-stage (0 to II) histologically confirmed lesions [32]. In this study, ultrasound technology was used to assess tumor depth, guiding the initial RT regimen and subsequent dosage adjustments, likely contributing to the high control rate. The superiority of image-guided RT over non-image-guided RT (both external and superficial) was further confirmed by comparative analysis, and patients undergoing image-guided RT generally tolerated the procedure well, with the majority (78.9%) experiencing only mild erythema and dry exfoliation [33]. There are several groups of patients that potentially benefit from SRT as a non-invasive treatment alternative. One important group consists of solid-organ-transplant recipients, who face a significantly higher risk of developing squamous cell carcinoma (SCC)—up to 250 times higher than the general population [34]. Interestingly, patients on mTOR inhibitors, such as sirolimus and everolimus, may exhibit increased radiosensitivity in their cancer cells, which, in turn, would make them more suitable candidates for image-guided hypofractionated or ultra-hypofractionated radiotherapy as a definitive treatment [35]. Other groups that may benefit from SRT are geriatric populations and those with severe comorbidities for whom surgical procedures are preferably discouraged.

This body of evidence not only supports our findings regarding the potential of SRT as a treatment for NMSC but also highlights the efficacy of using non-invasive imaging techniques, such as RCM, for monitoring treatment response.

## 5. Conclusions

In conclusion, our study demonstrates the successful non-invasive management of localized, limited-stage NMSC, including diagnosis, therapy, and surveillance. With additional training in non-invasive diagnostic and interventional techniques, there is potential to shift from a blanket surgical approach to a more patient-centered management strategy for skin cancers. To further validate the effectiveness of RCM in monitoring SRT for NMSC treatment, studies with longer follow-up periods and larger study populations are needed.

## Figures and Tables

**Table 1:** Patient characteristics. NMSC = non-melanoma skin cancer. SCC = squamous cell carcinoma. SCCis = SCC in situ. BCC = basal cell carcinoma. N = absolute number.

Characteristics	N (%)
Total patients	29
Total lesions	38
Sex Females Males	9 (31%) 20 (69%)
Age (mean ± SD) in years Females Males	70.5 ± 12.4 71.9 ± 17.2 69.9 ± 10
Ethnicity Caucasian Hispanic	28 (97%) 1 (3%)
Patients with history of NMSC	25 (86%)
Type of NMSC (histology) Total SCC SCCis BCC Nodular Superficial	38 (100%) 24 (63%) 6 (16%) 14 (37%) 10 (26%) 4 (11%)
Tumor location Scalp Face Neck Trunk Upper extremity Lower extremity	4 (10.5%) 14 (37%) 2 (5%) 2 (5%) 15 (39.5%) 1 (3%)

**Table 2:** Treatment outcome at 6-month follow-up as determined via reflectance confocal therapy (RCM). Patients that were lost to follow-up are excluded. N = absolute number.

Features Observed via RCM	N (%)
Total lesions *(patients lost to follow-up are excluded)*	32 (100%)
Residual tumor activity	0
Scar tissue	32 (100%)
Inflammation None Mild Moderate Severe	14 (44%) 12 (37.5%) 4 (12.5%) 2 (6%)

## Data Availability

The dataset used is available on request from the authors.
